# Evaluation of Ceramic Membrane Filtration for Alternatives to Microplastics in Cosmetic Formulations Using FlowCam Analysis

**DOI:** 10.3390/membranes15010035

**Published:** 2025-01-19

**Authors:** Seung Yeon Kim, Soyoun Kim, Chanhyuk Park

**Affiliations:** Department of Environmental Science and Engineering, Ewha Womans University, Seoul 03760, Republic of Korea

**Keywords:** alternative microplastics, ceramic membrane, cornstarch, fluid imaging microscopy, silica particle

## Abstract

The rapid expansion of the cosmetics industry has significantly increased the adoption of alternative microplastics in response to increasingly stringent global environmental regulations. This study presents a comparative analysis of the treatment performance of silica powder and cornstarch—common alternatives for microplastics in cosmetics—using ceramic membrane filtration combined with flow imaging microscopy (FlowCam) to analyze particle behavior. Bench-scale crossflow filtration experiments were performed with commercially available alumina ceramic membranes. By analyzing high-resolution images from FlowCam, the transport and retention behaviors of the two microplastic alternatives were examined by comparing their morphological properties. Despite their similar particle sizes, the cornstarch demonstrated a higher removal efficiency (82%) than the silica (72%) in the ceramic membrane filtration due to its greater tendency to aggregate. This increased tendency for aggregation suggests that cornstarch may contribute to faster fouling, while the stability and uniformity of silica particles result in less fouling. The FlowCam analysis revealed that the cornstarch particles experienced a slight increase in circularity and compactness over time, likely due to physical swelling and aggregation, while the silica particles retained their shape and structural integrity. These findings highlight the impact of the morphological properties of alternative microplastics on their filtration behavior and fouling potential.

## 1. Introduction

Microplastics, defined as plastic particles with diameters of ≤5 mm, are categorized into two types: primary and secondary [1,2,3]. Primary microplastics are intentionally manufactured, often as microbeads used in cosmetics, cleansers, and personal care products [4,5,6]. Examples include polyethylene (PE), polypropylene (PP), polyethylene terephthalate (PET), polymethyl methacrylate (PMMA), and nylon [4,5], which are valued for their physical and cosmetic benefits in exfoliants and cleansers. However, their release raises significant ecological concerns. Secondary microplastics, on the other hand, result from the degradation of larger plastic products through physical, chemical, and biological processes [7,8,9,10]. Growing awareness of microplastics’ environmental impact has prompted many countries to implement regulations that limit their use in cosmetics [11,12]. The United States and the European Union have spearheaded these efforts, introducing bans on the distribution of microbead-containing cosmetics to mitigate their environmental impact [13,14]. These regulations also apply to other consumer products containing microbeads, contributing to a significant reduction in microplastic release [11,14,15]. Consequently, the cosmetics industry has been compelled to develop alternative ingredients that retain product performance while minimizing environmental harm [12,13,16]. These alternatives must replicate the physical and chemical properties of microplastics while reducing the ecological impact [12,17,18].

Cornstarch, composed of the polysaccharides amylose and amylopectin, offers distinct functionalities in cosmetic formulations as a microplastic alternative. Amylose, a linear polymer with low water solubility, contributes to gel formation and stabilizes texture, enhancing the structural integrity of products. Amylopectin, a highly branched polymer with greater solubility and swelling capacity, ensures smooth application, rapid absorption, and a soft, non-sticky finish, making it essential for improving sensory qualities. Tricalcium phosphate (β-TCP) acts as an anti-caking agent, preventing particle aggregation and maintaining a free-flowing texture. Due to its natural origin, biodegradability, and non-toxic properties, cornstarch is increasingly used as a microplastic alternative. Its ability to form gels, absorb water, and improve texture makes it suitable for replacing microplastics in cosmetics, providing similar functionality while being environmentally friendly. Silica, in the form of silicon dioxide (SiO_2_), has emerged as a promising eco-friendly alternative to microplastics [16,19]. It functions effectively as an exfoliating agent, offering exceptional chemical resistance and thermal stability, which ensures reliable performance across diverse pH and temperature conditions [20]. Silica is non-toxic, causes minimal skin irritation, and provides a high level of user safety, making it an ideal replacement for microplastics [20]. Its high biodegradability and gentle texture make it particularly suitable for use in exfoliants and cleansing products, providing similar physical properties and functionality while significantly reducing environmental impacts [12,21].

These microplastic alternatives present significant challenges for removal in wastewater treatment facilities due to their small size and irregular morphology [22,23], highlighting the urgent need for effective, tailored treatment technologies. Advanced oxidation processes, such as UV- and ozone-based treatments with catalysis, are often ineffective, as they can break down particles into smaller, potentially more toxic fragments [24,25,26,27]. In contrast, membrane-based treatments offer a promising solution, relying on physical separation without generating harmful by-products [8,28,29,30]. In recent years, ceramic membranes, which are composed of materials such as alumina, silicon carbide, titania, and zirconia, have emerged as particularly effective alternatives to traditional polymeric membranes [31,32,33]. Their superior hydrophilicity and enhanced water permeance make them ideal for addressing the challenges posed by microplastic alternatives in wastewater treatment.

This study examines two alternative cosmetic ingredients (cornstarch and silica) using fluid imaging flow cytometry (FlowCam) for the automated analysis of microplastic alternatives. FlowCam enables the precise quantification of total particle count and the rapid morphological characterization of individual particles by analyzing images from liquid samples [34,35,36]. Compared to traditional methods, such as µ-FTIR and µ-Raman spectroscopy, which are limited to particles larger than 20 µm and 10 µm, respectively, and require significant time to analyze smaller particles [37,38], FlowCam offers faster processing across a broader range of particle sizes [24,39]. This capability facilitates a detailed evaluation of the behavior of cornstarch and silica during ceramic membrane filtration.

This study aimed to thoroughly characterize the physicochemical properties of two distinct microplastic alternatives—cornstarch and silica—to understand their transport and retention mechanisms during ceramic membrane filtration. This research evaluated filtration and treatment performance by analyzing particle behavior before and after passing through a commercially available tubular ceramic membrane, employing FlowCam analysis to identify potential treatment technologies for these microplastic alternatives. Furthermore, we systematically assessed the impact of these alternatives on membrane fouling, offering insights into optimizing operating conditions for efficient long-term applications.

## 2. Materials and Methods

### 2.1. Microplastic Alternatives

In this study, cornstarch (Johnson’s^®^ Cornstarch Baby Powder, Johnson & Johnson, New Brunswick, NJ, USA) and silica powders (Zero Sebum Drying Powder, ETUDE Corp., Seoul, Republic of Korea) were used as microplastic alternatives in cosmetic ingredients. Each alternative was dissolved in deionized (DI) water (Direct-Q^®^ 3 Water Purification System, Millipore Corp., Billerica, MA, USA) to create feed solutions at various concentrations. The feed concentrations of both microplastic alternatives were prepared at 50, 100, 150, 300, and 450 mg/L, and their total suspended solids (TSS) concentrations were subsequently measured. When the cornstarch solution was prepared at 264 mg/L and the silica powder solution at 300 mg/L, both solutions exhibited the same TSS concentration after calibration.

The cornstarch powder comprises 99.24% cornstarch, tricalcium phosphate (β-TCP), and a small amount of fragrance. According to the manufacturer, its particle size ranges from 10 to 100 µm, with a density of approximately 1.5–1.6 g/cm³. The silica (SiO_2_) present in the product has a crystalline structure composed of silicon and oxygen, with particle sizes ranging from nano-meters to micro-meters [40]. Its density is approximately 2.2–2.6 g/cm³, and it is virtually insoluble in water [40]. The product also contains mica (KAl_2_(AlSi_3_O_10_)(OH)_2_), which provides a shiny appearance, hydroxyapatite (Ca_10_(PO_4_)_6_(OH)_2_), and triglycerides derived from caprylic and capric acids (C_12_H_24_O_10_) for moisturizing benefits.

To assess the size characteristics of these microplastic alternatives, a particle size distribution analysis was performed using the Mastersizer 3000 (Malvern Panalytic Ltd., Malvern, UK). This instrument employs laser diffraction to ensure the proper dispersion and dilution of the prepared samples. The measurement range and mode were configured using the instrument’s software, which automatically collected data on particle size distribution. Key size parameters, including d_10_, d_50_, and d_90_, representing the particle sizes at which 10%, 50%, and 90% of the particles are finer, respectively, were measured. TSS was quantified by filtering 50 mL of the sample through 47 mm GF/C filters (Whatman^®^ Inc., Maidstone, UK) followed by drying the residue at 110 °C, in accordance with Standard Method 2540 D/E. The solubility of the microplastic alternatives was determined by subtracting the mass of the TSS (M_TSS_) in a solution from the total mass of the sample (M_total_). The solubility (S_dissolved_) was then calculated using the following formula:

$$S_{dissolved}=\frac{M_{total}-M_{TSS}}{V}$$

where V is the volume of the sample in liters.

### 2.2. Ceramic Membrane Filtration System

**Figure 1:**
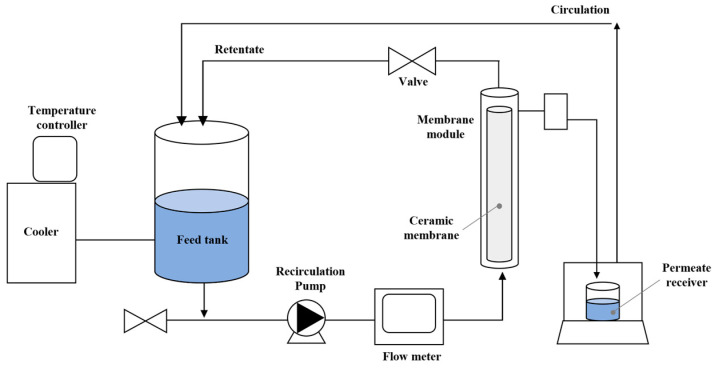
Schematic of the crossflow tubular ceramic membrane filtration system.

A commercially available alumina (Al_2_O_3_) ceramic microfiltration (MF) membrane (T1-70, Membralox^®^, Pall Corp., Deland, FL, USA) was used to characterize the transport and retention behavior of microplastic alternatives for achieving efficient separation. The membrane featured a 0.1 μm pore size, an inner diameter of 7 mm, a length of 250 mm, and an effective surface area of 50 cm^2^ (Table 1). It was housed in a stainless-steel module containing a single-channel Membralox^®^ T1-70 ceramic membrane, securely sealed with O-rings protected by washers and compressed using threaded Teflon-lined plugs. A schematic of the crossflow ceramic membrane filtration setup is shown in Figure 1. The system operated in inside-out mode and comprised a filtration module, a feed reservoir with an automatic temperature controller, a pump, piping, and other essential components for evaluating filtration performance. Experiments were conducted independently for each microplastic alternative at a constant transmembrane pressure (TMP) of 1.0 bar, regulated by a back-pressure regulator.

Before each experiment, the ceramic membrane was immersed in DI water for 24 h to achieve stabilization and uniform hydration, thereby enhancing initial permeability and ensuring consistent filtration conditions. The feed tank, equipped with a cooling system and an automatic temperature controller, maintained the feed solution at a stable ambient temperature (25.8 ± 0.6 °C) to minimize temperature-induced viscosity fluctuations. Approximately 3.5 L of the feed solution containing microplastic alternatives was added to the tank, which was then covered with a lid to prevent contamination. The feed solution was recirculated in a closed-loop batch operation mode, utilizing a pump controlled by a frequency converter to maintain a consistent flow rate. Permeate was collected in a glass beaker, and its weight was measured at regular intervals using an electronic scale, enabling real-time monitoring of permeate flux. Upon completion of each filtration test, the membrane was flushed with DI water to remove residual particulates and other components. It was then chemically cleaned with 500 mg/L sodium hypochlorite (NaOCl) for 1 h, followed by a 30 min rinse with DI water to remove residual cleaning agents. Pure water permeability was measured before each experiment to confirm water flux recovery and ensure membrane cleaning efficiency. The cleaning efficiency was assessed by measuring pure water permeability after each cleaning procedure. During this process, pure water was filtered for 20 s, and the permeate volume was recorded. Cleaning was considered successful if the permeate volume consistently reached 30 mL/20 s over three consecutive trials. This procedure ensured consistent water flux recovery before each experiment and validated the cleaning protocol used in this study.

### 2.3. Ceramic Membrane Characterization

The surface morphology of the ceramic membranes was characterized using field-emission scanning electron microscopy (FE-SEM; Auriga, Carl Zeiss, Oberkochen, Germany) at an acceleration voltage of 15.0 kV, which provided detailed visualization of the membranes’ layered structures. The pore sizes of the membranes were determined by evaluating their retention performance for polyethylene oxide (PEO; Sigma–Aldrich, St. Louis, MO, USA) solutions with varying molecular weights. The molecular weight cut-off (MWCO), defined as the smallest molecular weight of organic solutes retained at 90%, was determined by filtering a 1.5 g/L PEO solution. Filtration experiments were performed at an applied pressure of 3 bar for 2 h using a bench-scale crossflow ceramic membrane filtration system. PEO retention was quantified by analyzing PEO concentrations in the feed and permeate solutions using the non-purgeable organic carbon (NPOC) method with a total organic carbon (TOC) analyzer (TOC–LCPH, Shimadzu Corp., Kyoto, Japan). The average pore size of the ceramic membranes was then calculated using Equation (1), which correlates molecular weight (MW, g/mol) to pore size (d_s_, nm) as described in a previous study [41].

d_s_ = 0.01044 × MW^0.587^ (1)

### 2.4. FlowCam Analysis

FlowCam analysis was performed to analyze microplastic alternatives using a bench-top dynamic particle imaging analyzer (FlowCam 8100, Yokogawa Fluid Imaging Technologies, Inc., Scarborough, ME, USA) integrated with VisualSpreadsheet^®^ software (version 5.9.1.78). The system employed field-of-view (FOV) flow cells, enabling a digital camera to capture the entire width of the flow cell for enhanced particle detection and accurate representation. VisualSpreadsheet^®^ software was used to operate the FlowCam, process samples, capture and crop images, extract particle parameters, and classify images semi-automatically with user-defined libraries and statistical pattern-recognition algorithms. Before operation, the device was calibrated using a 100 μm polystyrene bead standard solution (4 K100 Duke Standards™, Thermo Scientific™, Waltham, MA, USA), and the flow cell was cleaned with a low-foaming liquid acid cleaner (1% (*v*/*v*) Citrajet, Alconox Inc., New York, NY, USA), ethanol, and DI water, following manufacturer recommendations. The background intensity was set to 175 to differentiate microplastics from the background. Based on the particle size range of cornstarch and silica, a 10× magnification objective lens was selected, paired with a suitable FOV flow cell, and optimized for particles between 1 and 100 µm.

During the analysis, the prepared samples were injected at a controlled flow rate of 0.125 mL/min, with images captured in auto-image mode at 13 frames per second. Artefacts, such as dirt, contaminants, and bubbles, were filtered out to minimize the detection of non-target particles. The system demonstrated an efficiency of 78.1% over an 8 min runtime. To ensure reliability, all identification and classification processes were repeated three times. This setup enabled the acquisition of high-resolution digital images of the microplastic alternatives in the feed and permeate samples, providing detailed morphological data. Shape-based volumetric parameters, including area-based diameter (ABD) volume, equivalent spherical diameter (ESD) volume, and cylindrical biovolume, were determined from the FlowCam measurement outputs. ABD volume was calculated as the diameter of a circle with an area equivalent to the particle’s dark pixel area. ESD volume was derived from the average of equally spaced Feret measurements, representing the perpendicular distance between two parallel lines touching either side of the particle. Biovolume was estimated by modeling particles as cylindrical shapes. Morphological parameters, including fiber curl, straightness, and circularity, were also assessed. Fiber curl was calculated by dividing the geodesic length by the Feret-based length and subtracting 1. Fiber straightness was determined as the ratio of Feret-based length to geodesic length, with a value of 1 indicating a straight fiber and increasing complexity reducing this value to 0. Circularity, a measure of particle roundness, was calculated as the square of the circumference of the equivalent spherical area divided by the square of the measured perimeter.

## 3. Results and Discussion

### 3.1. Physicochemical Properties of Microplastic Alternatives and Ceramic Membrane

#### 3.1.1. Microplastic Alternatives

**Figure 2:**
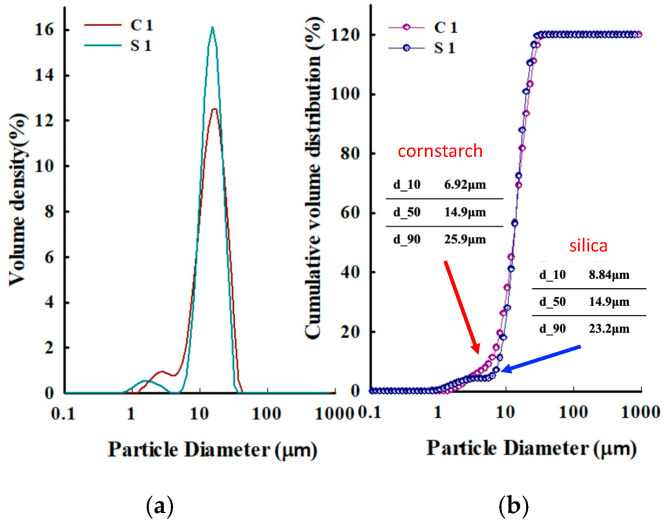
Volume-weighted particle size distribution curves of (**a**) cornstarch and (**b**) silica particles with an average size of 14.9 μm. The x-axis has a logarithmic scale.

The average particle size (d_50_) of both the cornstarch and silica particles was measured using a particle size distribution analyzer, with both recorded at 14.9 μm (Figure 2). However, their particle size distributions differed significantly. The cornstarch displayed a broader size distribution, with particle sizes ranging from very small to considerably larger, indicating higher heterogeneity. Conversely, the silica particles exhibited a narrower, more uniform distribution with minimal size variation. These differences significantly influence filtration behavior. The wider size distribution of the cornstarch particles, especially the presence of larger particles, may promote faster accumulation on the membrane surface and accelerate fouling. Conversely, the uniform size distribution of the silica particles facilitates more consistent transport through the membrane, potentially reducing fouling tendency. This indicates that the heterogeneity of cornstarch, particularly its larger particles, may contribute to more rapid pore blocking or surface accumulation during filtration.

#### 3.1.2. Ceramic Membrane

**Figure 3:**
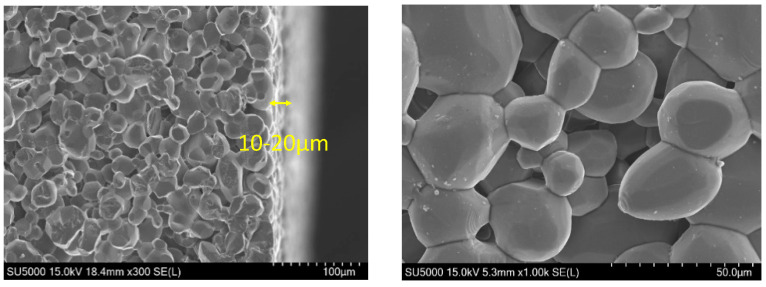
FE-SEM cross-sectional images of the Membralox^®^ tubular ceramic membrane.

Membralox^®^ ceramic elements, characterized by an asymmetric membrane structure, are engineered through a specialized design and manufacturing process that makes them highly suitable for applications involving chemicals such as solvents, extreme pH conditions, high temperatures, and pressure processing of fermentation broths. The structural and chemical properties of the ceramic membrane were comprehensively analyzed to elucidate its retention mechanism. Given its inside-out filtration mode, the inner (feed-side) surface of the tubular ceramic membrane was examined using FE-SEM (Figure 3). The structural analysis focused on the composition of the membrane’s surface layer and pore size, both of which significantly contribute to the treatment and filtration performance of the ceramic membrane. The FE-SEM cross-sectional images revealed an α-alumina (α-Al_2_O_3_) layer with a thickness of 10–20 µm, consistent with the chemical composition data provided by the manufacturer. Notably, the analysis showed no distinct boundary between the surface and support layers, with the membrane layers overlaying a more porous support layer.

The pore size of the Membralox^®^ tubular ceramic membrane was determined using capillary flow porometry and the bubble point test. The mean flow pore diameter was 0.0268 µm, while the bubble point diameter, representing the largest pore, was 0.0273 µm. The small difference of 0.0005 µm suggests a relatively uniform pore size distribution. The discrepancy between the measured pore sizes and the manufacturer’s nominal value of 0.1 µm is attributed to differences in the analytical methods used. The manufacturer’s value likely represents the overall pore structure, highlighting larger pores. However, capillary flow porometry specifically measures the active pores involved in fluid transport, excluding non-conductive or inactive pores. As a result, smaller average and maximum pore sizes are reported. Additionally, capillary flow porometry primarily focuses on surface pores that directly influence fluid flow, whereas the manufacturer’s nominal pore size reflects the average pore size across the entire membrane, including the internal layers. Finally, variations in the manufacturing process may result in actual pore sizes differing from the specified nominal value.

**Figure 4:**
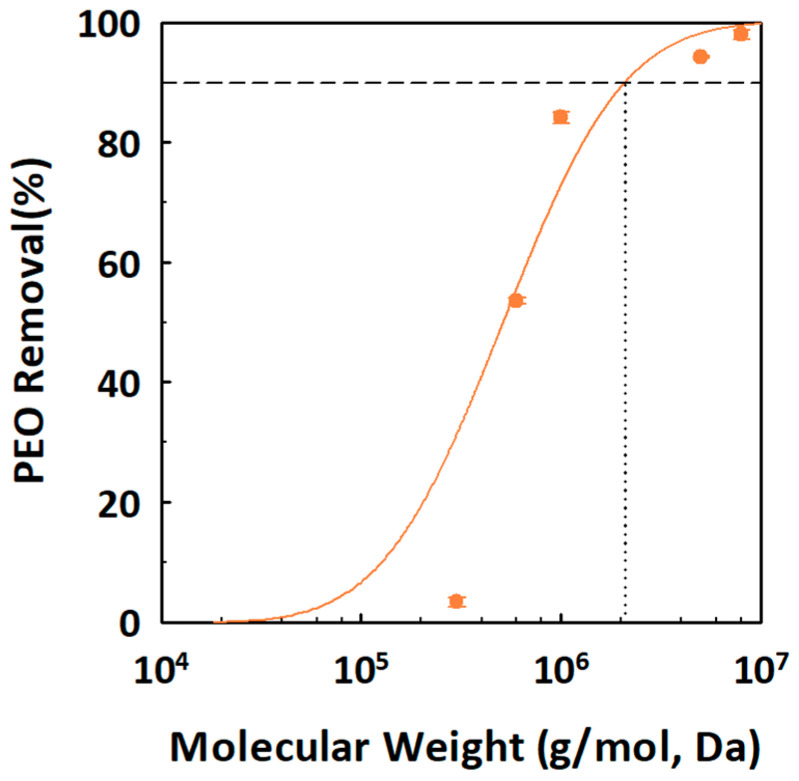
Cumulative distribution function for polyethylene glycol (PEO) solutions with molecular weights of 300–8000 kg/mol, filtered through a ceramic membrane. The fitted line presents the cumulative log-normal distribution function based on the results of the PEO retention experiment. Removal efficiency is based on the non-purgeable organic carbon concentration of the solutes.

To estimate the average pore size of the ceramic membrane, retention experiments were performed using PEO solutions with five distinct MWCO values. The cumulative log-normal distribution function for PEO retention, shown in Figure 4, indicates that PEO solutions with 90% retention efficiency correspond to an average pore size of about 53.5 nm, equivalent to an MWCO of approximately 2088 kDa (Equation (1)). Although this value is smaller than the manufacturer-specified pore size of 0.1 µm, it remains within the expected range for MF membranes, which typically have MWCOs above 1000 kDa [42]. This discrepancy may stem from variations in measurement methods or operating conditions. Nonetheless, the results confirm the ceramic membrane’s effective retention of PEG molecules, demonstrating its suitability for applications that require precise molecular separation.

### 3.2. Morphological Changes of Microplastic Alternatives During Filtration

To assess the retention behavior of the cornstarch and silica particles during ceramic membrane filtration, shape-related parameters including ABD, ESD, and biovolume were analyzed alongside morphological properties such as circularity, aspect ratio, and compactness using FlowCam on Days 1 and 3. The analysis examined the characteristics of microplastic alternatives over time and between the feed and permeate samples (expressed as the permeate-to-feed ratio, p/f). These variations provided critical insights into filtration efficiency and fouling mechanisms, facilitating a detailed comparison of the filtration behaviors of the two microplastic alternatives and their impacts on membrane fouling.

#### 3.2.1. Shape-Based Volumetric Parameters

Significant reductions in ABD, ESD, and biovolume were observed for cornstarch between Day 1 and Day 3 (Table 2). The p/f ratio for ABD decreased from 0.9157 on Day 1 to 0.6776 on Day 3. Similarly, the p/f ratio for ESD decreased from 0.8529 to 0.7216, while that for biovolume dropped from 0.8737 to 0.5749. These findings suggest that the cornstarch particles became progressively smaller and exhibited reduced volume after filtration. Furthermore, the standard deviations for ABD, ESD, and biovolume showed slight reductions from Day 1 to Day 3, reflecting increased uniformity in particle size over time. For example, the standard deviation of ABD declined from 0.0460 to 0.0271, indicating that prolonged exposure to water enhanced particle uniformity. This trend is attributed to the increased solubility of cornstarch in water, which promotes the formation of more homogeneous particles that are easier to filter.

The silica particles exhibited different transport behaviors compared to cornstarch during filtration. The p/f ratios for ABD, ESD, and biovolume decreased consistently from Day 1 to Day 3, indicating reductions in particle size and volume. Specifically, the p/f ratio decreased from 0.9251 to 0.7455 for ABD, from 0.8885 to 0.6441 for ESD, and from 0.9701 to 0.6936 for biovolume. The greater reduction in ESD compared to ABD suggests more pronounced morphological changes in the silica particles, such as elongation or irregularity, as evidenced by the FlowCam data. The standard deviations for both ABD and ESD also decreased, indicating increased uniformity in particle size and shape after filtration. For example, the standard deviation for ABD slightly declined from 0.0530 to 0.0511, while that for ESD decreased more significantly from 0.0352 to 0.0137. This trend suggests that filtration preferentially removed larger and more irregular particles, leaving a more uniform population of smaller, regular-shaped silica particles in the permeate.

#### 3.2.2. Morphological Parameters

The analysis of morphological parameters revealed distinct behaviors between the cornstarch and silica particles during filtration (Table 3). After three days of exposure to water, the cornstarch particles exhibited a more regular shape. Circularity slightly increased, likely due to the swelling and dissolution of larger particles into smaller fragments and potential aggregation during filtration, resulting in a more spherical shape. Compactness also increased, indicating particle agglomeration and the formation of a fouling layer on the membrane surface, making the cornstarch a significant contributor to fouling. Similarly, the silica particles exhibited slight changes in circularity and compactness while maintaining their chemical and physical stability. These particles retained their structural integrity throughout the filtration process, preventing substantial fouling layer formation and preserving mechanical consistency. Both microplastic alternatives showed increased circularity and compactness after filtration, suggesting that the membrane preferentially captured larger, more irregular particles, leaving behind smaller, more regular-shaped ones. These findings highlight the importance of particle morphology in filtration efficiency and fouling behavior, with each microplastic alternative exhibiting distinct characteristics due to its chemical and physical properties. The solubility and water absorption of cornstarch were key factors influencing its filtration performance, while the mechanical deformation of silica significantly altered its filtration properties after filtration [43].

### 3.3. Removal Efficiency of Microplastic Alternatives in Cosmetics

**Figure 5:**
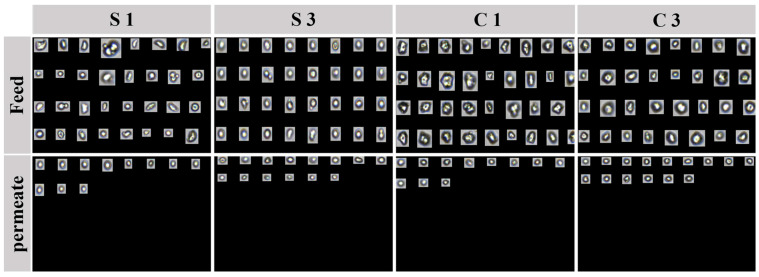
Representative images of cornstarch and silica powder as microplastic alternatives in feed and permeate samples after ceramic membrane filtration, analyzed using FlowCam.

The change in particle counts before and after ceramic membrane filtration provides critical insights into the removal efficiency of microplastic alternatives in cosmetics. This study employed FlowCam to analyze particle count variations for cornstarch and silica powders, facilitating a comparative evaluation of their removal efficiencies. Representative images of the microplastic alternatives, captured before and after ceramic membrane filtration, are presented in Figure 5. The FlowCam setup was optimized to reduce image duplication and enhance the accuracy of particle count measurements. Measurements were performed at 10× magnification, enabling detailed observation of particle morphology and behavior. The flow rate was maintained at 0.125 mL/min to ensure steady sample movement through the system, and imaging was conducted at an auto-capture rate of 13 frames per second to obtain high-resolution data. Following image capture, a thorough filtering process was applied to remove noise, such as bubbles or non-particle artifacts, ensuring both quantitative and qualitative accuracy. The filtering criteria used for analysis are provided in Table S1. These criteria were carefully selected to emphasize the physical attributes of the particles, filter out irregular objects, and ensure that only relevant microplastic alternatives were included in the analysis. This filtering procedure facilitated the identification of actual microplastic alternatives based on their geometry and optical characteristics, establishing a robust protocol for accurate and reproducible particle tracking and analysis.

**Figure 6:**
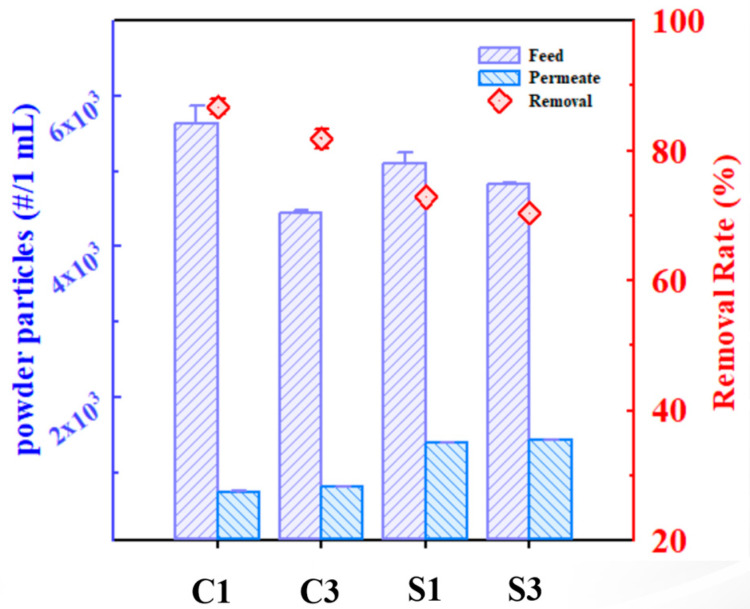
Total number of quantified particles of cornstarch and silica, stirred in deionized water for one and three days, in both feed and permeate samples (1 mL) after ceramic membrane filtration.

Based on the high-accuracy filtered data, the cornstarch achieved a removal efficiency of 82%, while the silica powder reached 72%, indicating that the cornstarch was more effectively removed by the ceramic membrane (Figure 6). This difference highlights the impact of their distinct physicochemical properties during filtration. The organic, polysaccharide-based structure of cornstarch facilitated aggregation and removal, whereas the inorganic nature of silica limited its interaction with water, resulting in lower removal efficiency. The size and distribution analysis presented in Figure 2 further confirms that the tendency of cornstarch to form larger aggregates over time significantly contributed to its higher removal efficiency. Conversely, the silica particles exhibited minimal changes in size distribution due to their lower aggregation tendency, resulting in reduced removal efficiency. Both the cornstarch and silica powder showed slight variations in particle count over time after filtration. While the reported average particle sizes of the two microplastic alternatives are larger than the nominal pore size of the membrane, the average size does not represent the entire particle population. Both substances exhibit a wide particle size distribution, with smaller particles constituting a significant proportion. These smaller particles are more likely to pass through the membrane pores, resulting in a lower overall rejection. The membrane does not have a uniform pore size and contains pores larger than the nominal pore size. These larger pores may allow the passage of the particles that would typically be retained. During the filtration process, the applied pressure can deform or compress certain particles, reducing their effective size and enabling them to pass through the membrane pores [22]. This is particularly relevant for substances like cornstarch, which may exhibit compressibility under pressure. Hydrodynamic interactions between particles and the membrane surface, especially at high operating pressures, can also contribute to particle transport through the membrane. These interactions may facilitate the passage of particles that would otherwise be too large to pass through the nominal pore size. Notably, the cornstarch demonstrated a more significant reduction in particle count, with a 21.1% decrease, compared to the silica (5.7%). This greater reduction in cornstarch particles is likely due to their natural tendency to aggregate in water, which enhances removal efficiency during filtration. The gel-forming and hygroscopic properties of cornstarch promoted the formation of larger particle clusters, which were more readily captured by the membrane. In contrast, silica’s lower aggregation tendency in water resulted in fewer particle clusters and, consequently, lower removal efficiency. The reduced aggregation of the silica particles contributed to their lower filtration efficiency, as smaller, more uniform particles passed through the membrane more easily.

### 3.4. Effect of Microplastic Alternatives on Membrane Fouling

**Figure 7:**
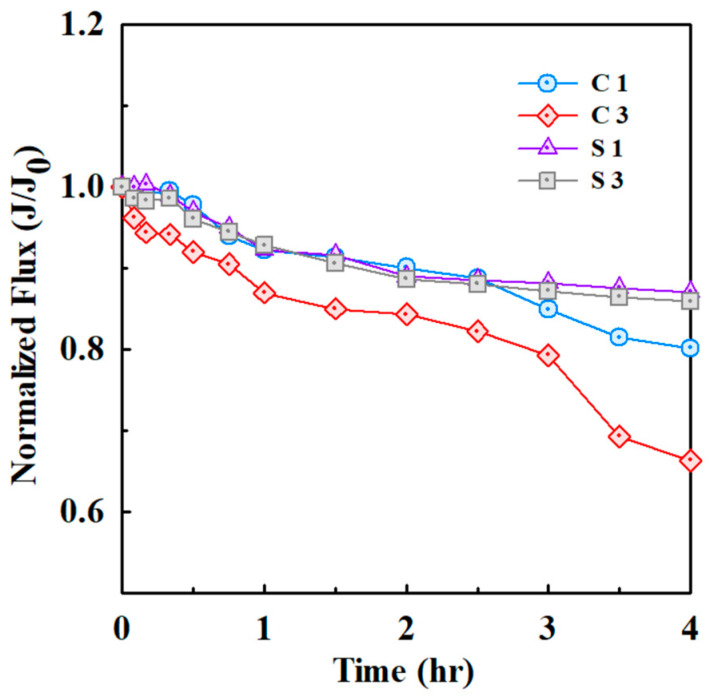
Normalized water flux decline for cornstarch and silica particles (with an average size of 14.9 μm) during ceramic membrane filtration.

The comparison of fouling behavior between the cornstarch and silica revealed that the cornstarch induced membrane fouling more rapidly than the silica (Figure 7). In a similar study, physically reversible resistance was identified as the primary form of resistance for ceramic MF membranes, primarily due to cake layer formation, which could be easily removed through washing [31]. This rapid membrane fouling is primarily attributed to the physical structure and chemical composition of cornstarch [44]. Composed of polysaccharides, cornstarch swells upon interacting with water, which increases its viscosity [45]. The density of cornstarch can vary depending on the measurement method, typically ranging from 1.45 to 1.60 g/cm^3^. Additionally, the swelling power of cornstarch increases with temperature and time [46]. This swelling causes the cornstarch particles to occlude membrane pores, thereby promoting fouling [47]. Additionally, water absorption leads to gelation, wherein polymer chains form hydrogen bonds with water, further increasing viscosity and contributing to the accumulation of viscous materials on the membrane surface [48,49]. This process significantly increases filtration resistance, thereby reducing permeate flux and shortening membrane lifespan [50]. These effects are corroborated by observed changes in solubility over time. Both the cornstarch and silica exhibited increased solubility over time; however, the cornstarch demonstrated a significantly faster rate of increase (Figure S1). This rapid solubility increase is attributed to its hydrophilic nature, which enhances viscosity and resistance during filtration [51,52]. In contrast, the silica, a chemically inert and stable material [45], exhibited slower fouling formation. Its negatively charged surface limits particle adhesion and aggregation, enabling it to pass through the membrane more easily [47,48]. Consequently, the silica experienced less physical fouling than the cornstarch, maintaining stable filtration performance. In summary, the rapid fouling observed with the cornstarch stems from its gelation properties, whereas silica’s stability and minimal interaction with water contribute to its slower fouling rate. This comparison highlights the importance of understanding the behavior of microplastic alternatives in optimizing filtration and extending membrane lifespan.

## 4. Conclusions

This study comprehensively evaluated the physicochemical and morphological properties of representative microplastic alternatives used in cosmetics and their impacts on ceramic membrane filtration performance. Filtration experiments revealed that the cornstarch achieved a higher removal efficiency (82%) than the silica (72%). This difference is attributed to the polysaccharide-based structure of cornstarch, which promotes aggregation and gelation in aqueous solutions, forming larger aggregates that are more readily captured by the membrane. However, the removal efficiency of the cornstarch decreased significantly over time, primarily due to its gradual dissolution in the feed solution. Conversely, the silica exhibited stable removal efficiency over time, with its inert inorganic nature and uniform particle size distribution contributing to lower aggregation tendencies, slower fouling rates, and consistent long-term filtration performance. The cornstarch’s high solubility and polysaccharide composition led to accelerated pore clogging and viscosity-related fouling, causing more severe fouling than the silica. The cornstarch particles showed reduced size (ABD, ESD, and biovolume) and increased uniformity, whereas the silica particles remained structurally stable, with the observed changes primarily attributed to mechanical deformation. Both materials showed increased circularity, suggesting that the ceramic membrane preferentially captured larger and more irregular particles. In summary, although the cornstarch exhibited higher removal efficiency, its rapid dissolution and significant fouling potential present challenges to sustained filtration performance. The silica, with its lower fouling tendency and stable structural properties, emerged as a more reliable option for long-term filtration applications. Future research should optimize filtration operating conditions to minimize fouling, refine cleaning protocols to enhance flux recovery, and explore scalable ceramic membrane coatings to improve filtration performance and durability.

## Figures and Tables

**Table 1:** Physical properties of the ceramic membrane used in this study.

Physical Properties	Specification
Manufacturer	T1-70, Membralox^®^, Pall Corp.
Pore size	0.1 μm
Active layer	α-alumina (α-Al_2_O_3_)
Inner and outer diameter	7 mm and 10 mm
Length	250 mm
Effective surface area	50 cm^2^

**Table 2:** Permeate-to-feed ratios for area-based diameter (ABD) volume, equivalent spherical diameter (ESD) volume, and biovolume of quantified cornstarch and silica particles.

Fraction (Permeate/Feed)	ABD Volume		ESD Volume		Biovolume	
	Day 1	Day 3	Day 1	Day 3	Day 1	Day 3
Cornstarch	0.9157 ± 0.12	0.6776 ± 0.18	0.8529 ± 0.14	0.7216 ± 0.16	0.8737 ± 0.21	0.5749 ± 0.19
Silica	0.9251 ± 0.07	0.7455 ± 0.09	0.8885 ± 0.05	0.6441 ± 0.07	0.9701 ± 0.11	0.6936 ± 0.09

**Table 3:** Permeate-to-feed ratios for circle fit, circularity, and compactness of quantified cornstarch and silica particles.

Fraction (Permeate/Feed)	Circle Fit		Circularity		Compactness	
	Day 1	Day 3	Day 1	Day 3	Day 1	Day 3
Cornstarch	1.0194 ± 0.23	1.0833 ± 0.31	1.0121 ± 0.19	1.0533 ± 0.42	1.0346 ± 0.33	1.0637 ± 0.38
Silica	1.0714 ± 0.09	1.1245 ± 0.12	1.0230 ± 0.16	1.0741 ± 0.19	1.0328 ± 0.35	1.0717 ± 0.24

## Data Availability

The original contributions presented in this study are included in the article/Supplementary Materials. Further inquiries can be directed to the corresponding author(s).

## Supplementary Materials

The following supporting information can be downloaded at: https://www.mdpi.com/article/10.3390/membranes15010035/s1, Figure S1: Time-dependent changes in solubility of cornstarch and silica powders under continuous stirring in distilled water; Table S1: Filter value settings for various parameters of captured images in this study.
