# Supplementary material for: Evaluation of Ceramic Membrane Filtration for Alternatives to Microplastics in Cosmetic Formulations Using FlowCam Analysis
Source: Membranes (Basel). 2025 Jan 19;15(1):35. doi: 10.3390/membranes15010035 (PMC11767035; doi:10.3390/membranes15010035)
Supplement: Supplementary file 1 [file membranes-15-00035-s001.zip › membranes-3387863-supplementary.pdf]

Supporting Information

Evaluation of ceramic membrane filtration for microplastic alternatives in cosmetic ingredients using FlowCam analysis

Seung Yeon Kim, Soyoun Kim and Chanhyuk Park*

Department of Environmental Science and Engineering, Ewha Womans University, Seoul 03760, South Korea

Submitted to

Membranes

*Corresponding author.

E-mail: chp@ewha.ac.kr (Chanhyuk Park)

Table S1. Filter value settings for various parameters of captured images in this study

Parameters	Diameter	Circularity	Intensity	Average green	Average blue
Filter value settings	MIN 5	MIN 0.3	MAX 130	MAX 155	MAX 160

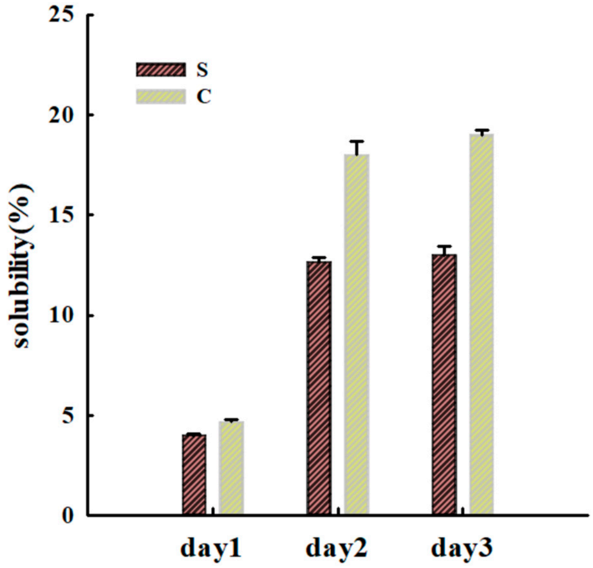

Figure S1 Time-dependent changes in solubility of cornstarch and silica powders under continuous stirring in distilled water.
